# Cultivation of human centered artificial intelligence: culturally adaptive thinking in education (CATE) for AI

**DOI:** 10.3389/frai.2023.1198180

**Published:** 2023-12-01

**Authors:** Yana Samuel, Margaret Brennan-Tonetta, Jim Samuel, Rajiv Kashyap, Vivek Kumar, Sri Krishna Kaashyap, Nishitha Chidipothu, Irawati Anand, Parth Jain

**Affiliations:** ^1^Middlesex County College, Edison, NJ, United States; ^2^Rutgers, The State University of New Jersey, New Brunswick, NJ, United States; ^3^William Paterson University, Wayne, NJ, United States; ^4^University of Cagliari, Cagliari, Sardinia, Italy

**Keywords:** human centered artificial intelligence, education, culture, AI, AI education, educational AI, culturally responsive teaching, AI philosophy

## Abstract

Artificial Intelligence (AI) has become ubiquitous in human society, and yet vast segments of the global population have no, little, or counterproductive information about AI. It is necessary to teach AI topics on a mass scale. While there is a rush to implement academic initiatives, scant attention has been paid to the unique challenges of teaching AI curricula to a global and culturally diverse audience with varying expectations of privacy, technological autonomy, risk preference, and knowledge sharing. Our study fills this void by focusing on AI elements in a new framework titled Culturally Adaptive Thinking in Education for AI (CATE-AI) to enable teaching AI concepts to culturally diverse learners. Failure to contextualize and sensitize AI education to culture and other categorical human-thought clusters, can lead to several undesirable effects including confusion, AI-phobia, cultural biases to AI, increased resistance toward AI technologies and AI education. We discuss and integrate human behavior theories, AI applications research, educational frameworks, and human centered AI principles to articulate CATE-AI. In the first part of this paper, we present the development a significantly enhanced version of CATE. In the second part, we explore textual data from AI related news articles to generate insights that lay the foundation for CATE-AI, and support our findings. The CATE-AI framework can help learners study artificial intelligence topics more effectively by serving as a basis for adapting and contextualizing AI to their sociocultural needs.

## Introduction

“It is crucial to make learning authentic and contextualize it in the lives and cultures of students so that it becomes meaningful for them. Especially with the task of teaching AI and ethics, … contextualization of the materials and topics used in curriculum help them make sense…”

Eguchi et al., 2021.

**Figure 1:**
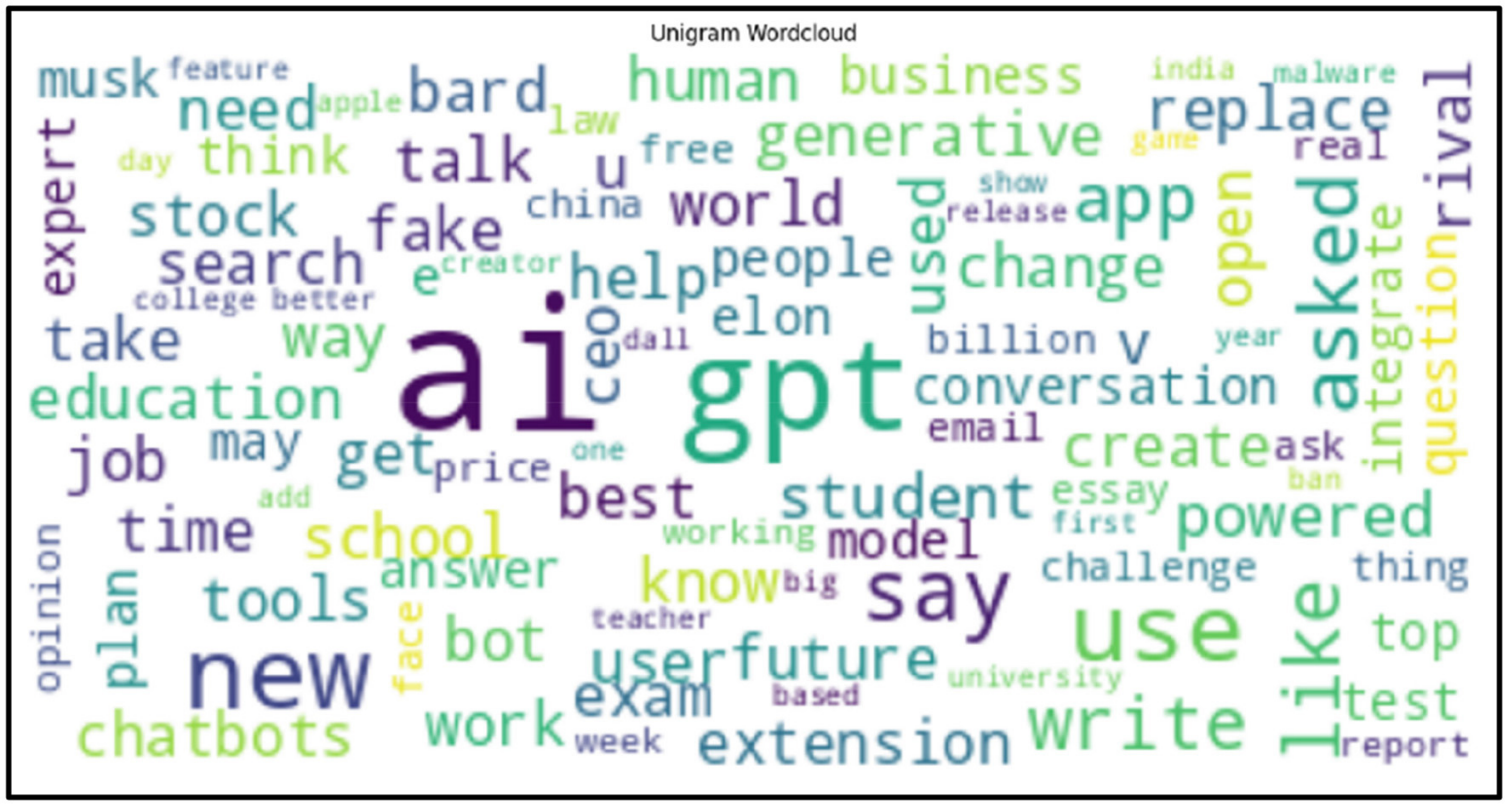
Word cloud of AI-ChatGPT News Articles.

Interest in artificial intelligence (AI) has peaked since November 2022 when OpenAI released ChatGPT. AI has escaped the confines of expert discussions, labs, devices, and technology applications and to emerge as a driver of mainstream societal progress in various domains such as education, healthcare and finance (King, 2022; Dowling and Lucey, 2023; Rudolph et al., 2023). AI technologies have rapidly invaded news media and public conversations and our analysis explored over forty thousand articles and posts (Figure 1). Our analysis covered media mentions of AI, natural language processing (NLP) and large language models (LLMs) in the media since 2022. Despite lack of knowledge, the appeal of generative AI has significantly increased public confidence in artificially intelligent capabilities. In the absence of rapid intervention and public education, this might lead to overconfidence and an over-reliance on AI tools like ChatGPT. The absence of sufficient sound conceptual and theoretical discussions on AI, human interaction with AI and AI education presents a compelling need to explore relevant conceptual frameworks.

AI and its subfield natural language processing (NLP) have become increasingly prevalent in every aspect of our lives, with public engagement intensifying since the advent of OpenAI's Generative Pretrained Transformers (GPT) based ChatGPT application (Wei et al., 2022). It is important to note that ChatGPT, a language model developed by OpenAI that uses machine learning to generate human-like responses to text prompts, functions largely as an opaque blackbox. Given that “*every human will interact with AI in the visible future, directly or indirectly, some for creating products and services, some for research, some for government, some for education, and many for consumption*,” it is vital to understand AI education (Samuel et al., 2022a). Consequently, we have witnessed an increase in AI curriculum, courses, programs, and academic efforts (Touretzky et al., 2019; Chiu et al., 2021; Samuel, 2021b). It is imperative to evolve AI education, as every individual will inevitably engage with artificial intelligence (AI) in the foreseeable future.

AI powered applications released within the past year such as BigScience Large Open-science Open-access Multilingual (BLOOM), Large Language Model Meta AI (LLaMA), Pathways Language Model (PaLM) and OpenAI (Google, 2023; HuggingFace, 2023; Meta, 2023; Open AI, 2023) represent the next wave of innovations that will help reimagine and reshape the future of the human race. These foundation models have been accompanied by pathbreaking AI research, which portend greater productivity per worker and enormous socioeconomic impacts. We assert that these advancements only serve to underscore the critical need to address the challenges of AI education.

Widespread ignorance about AI technologies and their global ramifications has rendered society unprepared for the impending AI wave. AI education must be re-envisioned so that future generations can deploy AI technologies responsibly and comprehend their potential consequences. AI education that emanates from the global North often encounters resistance due to fear of unintended consequences. However, to protect against the prospect of AI supremacy over humans (Samuel, 2022), we must step up efforts to spread AI education across the world.

Since AI education does not occur in a vacuum, each person's cultural background determines her or his ability to absorb AI instruction. AI education must be globally facilitated to ensure inclusiveness and equity, and locally contextualized to assure sensitivity to the needs of people of all ages, genders, races and cultures.

**Figure 2:**
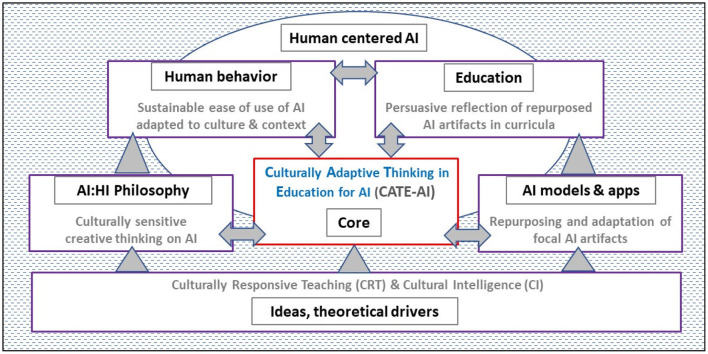
Culturally adaptive thinking in education for artificial intelligence (CATE-AI) framework.

AI innovations, AI education, and AI technologies are interrelated and interdependent. Our research unifies the most significant themes to provide a framework that we refer to as Culturally Adaptive Thinking in Education for AI (CATE-AI) as presented in Figure 2. CATE-AI provides a lens to focus AI education through cultural adaptivity, which embodies sensitivity to gender, ethnic, and age-based needs. Our exploratory analysis precludes hypotheses development and empirical tests. Instead, we employ inductive reasoning based on previous research, current and emerging technology trends, and cases drawn from news media.

**Figure 3:**
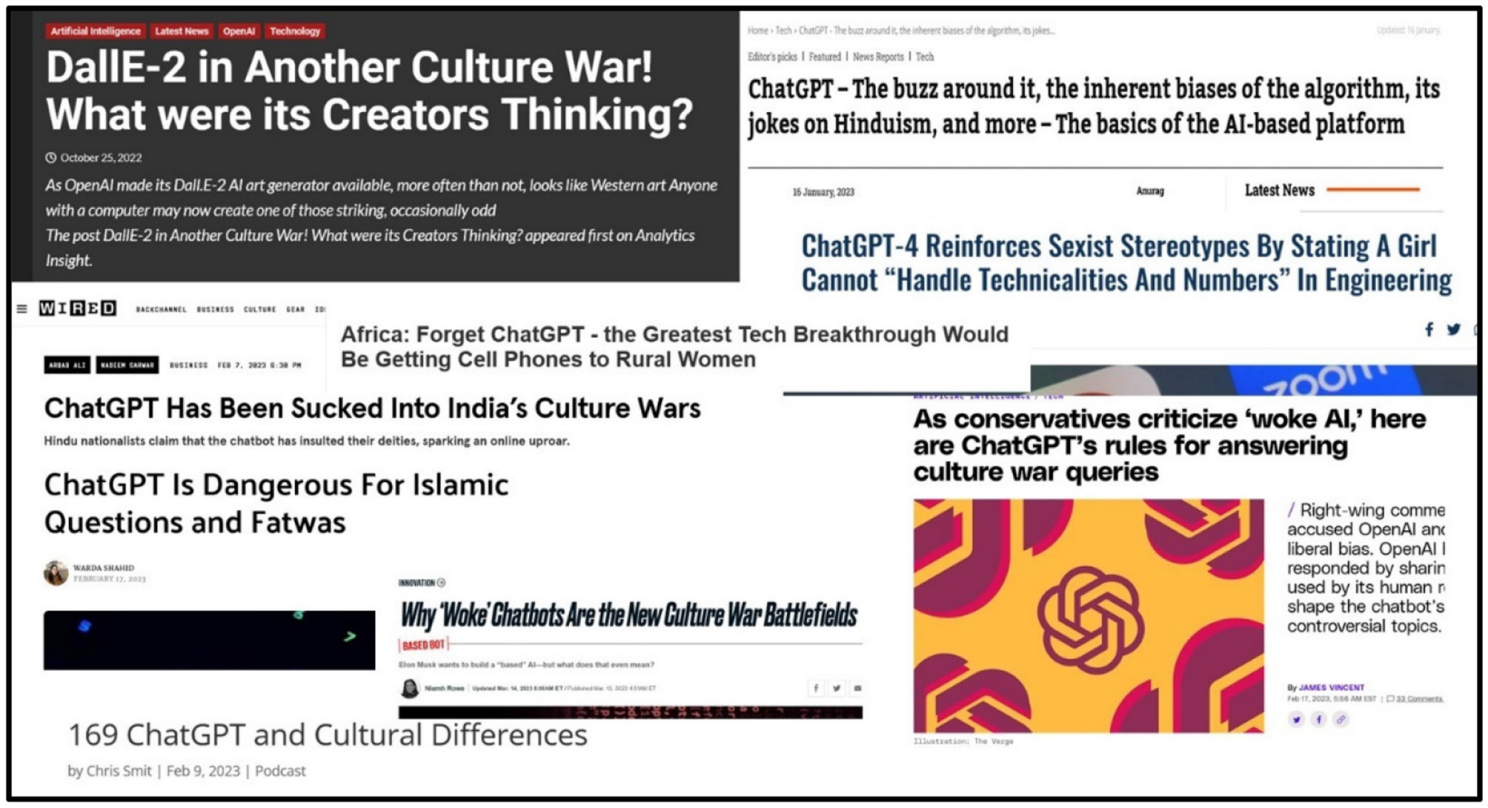
Need for adaptation of AI Tools and AI education evidenced globally. Sources for articles displayed in Figure, all accessed in March 2023: (1) https://www.equalitynow.org/news_and_insights/chatgpt-4-reinforces-sexist-stereotypes/; (2) https://allafrica.com/stories/202303070096.html; (3) https://www.opindia.com/2023/01/chatgpt-artifical-intelligence-chatbot-biased-here-is-how/; (4) https://www.thedailybeast.com/why-woke-chatbots-are-the-new-culture-war-battlefields; (5) https://www.theverge.com/2023/2/17/23603906/openai-chatgpt-woke-criticism-culture-war-rules; (6) https://theislamicinformation.com/news/chatgpt-dangerous-for-islamic-questions-fatwas/; (7) https://culturematters.com/chatgpt-and-cultural-differences/; (8) https://www.analyticsinsight.net/dalle-2-in-another-culture-war-what-were-its-creators-thinking/; (9) https://www.wired.com/story/chatgpt-has-been-sucked-into-indias-culture-wars/.

Our literature review elaborates and ties the relevant threads of research on AI, NLP, AI education, culturally responsive teaching, and cultural intelligence. This allows us to identify the unique needs of AI education and its challenges. Thereafter, we introduce our data, which consists of news headlines and illustrative examples of cultural bias of recent AI applications (Figure 3). Our findings from our exploratory NLP analysis demonstrate the need for increased cultural adaptivity. Next, we introduce and develop arguments for the CATE-AI framework (Figure 2), which is followed by a discussion of the limitations of the present study and ideas for future research. We make recommendations to enable the adoption of CATE-AI and conclude with a discussion of future research opportunities in AI education.

## Literature review

We review extant literature and use inductive reasoning to conceptualize the CATE-AI framework. The CATE-AI framework is built upon relevant research, AI and technology trends, and textual data from news media. We lay the foundation for the CATE-AI framework by drawing theories and concepts from the literature review. These include culturally responsive teaching and cultural intelligence, which help distinguish between AI education and educational AI. The adoption of a robust philosophy to understand the dynamics of interactions between humans and AI is a key tenet of CATE-AI. It serves as the crucible for melding the conceptual blocks that are the essence of CATE-AI.

### AI education is different from educational AI

Artificial intelligence in education is an ambiguous phrase that may be used to refer to AI curricula in education OR the use of AI technologies in education. We refer to the former as AI Education and the latter as Educational AI. In this paper, our primary focus is on AI Education and we clarify the difference. AI Education is the study of artificial intelligence, in the same way as mathematics education involves the study of mathematics. This means teaching the development of computer systems to perform tasks that typically require human intelligence, such as learning, problem-solving, perception, reasoning, and natural language processing. AI Education can be defined as the endeavors and objectives that are intrinsic to instructing and acquiring knowledge about artificial intelligence (Wollowski et al., 2016; Chen et al., 2020; Su et al., 2022; Ng et al., 2023).

In contrast, Educational AI denotes the utilization of artificial intelligence to enhance teaching and learning processes. This encompasses intelligent tutoring systems (Vanlehn, 2011; D'mello and Graesser, 2013), adaptive learning platforms (Muñoz et al., 2022), tools grounded in Natural Language Processing (NLP) (Chen et al., 2022; Shaik et al., 2022), chatbot applications (Paschoal et al., 2018), and learning analytics (Leitner et al., 2017).

Our research on AI Education is distinct from previous research on the use of AI technologies in education (i.e., Educational AI) (Chassignol et al., 2018; Tuomi, 2018). This paper focuses on contextualizing AI Education through cultural insights to facilitate access and adoption of Educational AI through fair and equitable processes.

### Human centered AI (HAI)

Understanding the impacts of diverse cultures on human behavior is of vital importance due to the growing emphasis on the social dimensions of human interactions in the context of virtual agents (Mascarenhas et al., 2016). Understanding AI capabilities and the dynamics of interactions between AIs and human intelligences is critical if we are to establish a foundation for Human-centered AI. AI capabilities are based on emulating human intelligence functions, particularly cognitive and logical processes (Samuel, 2021a). AI capabilities have allowed us to reshape conventions for interactions with machines, while disrupting diverse industries such as healthcare, manufacturing, music, media, and research. The ramifications are evident: AI's disruptive influence and reconfiguration of established social norms are propelling the advent of a fourth industrial revolution. Our interactions with machines have rapidly evolved due to the capacity of AI to replicate human decision-making. AI is poised to exert a substantial influence on our day-to-day lives as it will affect convenience, efficiency, personalization, privacy protection, and security (Feijóo et al., 2020). These changes are already underway, fundamentally reshaping our attitudes and dynamics of conduct.

The advancement of AI-driven technology marks the latest phase in our ongoing endeavor to automate human-performed tasks (Tai, 2020). While AI can be viewed as a continuation of innovations in automation, it stands apart from prior non-AI-driven technological progress. A fundamental distinction lies in how past scientific and industrial revolutions replaced human physical labor, whereas the current AI-led transformation has the potential to replace human intellectual capacities Samuel et al. (2022a). The potential of AI to substitute human intelligence has also prompted experts to differentiate between “weak AI,” focusing on specialized tasks, and “strong AI,” which emulates human cognitive functions. For example, while “weak AI” may excel in specific tasks like chess or equation solving, “strong AI” would surpass humans across a wide spectrum of cognitive activities (Lu et al., 2018).

Despite AI's swift progress and far-reaching societal effects, the global population remains largely uninformed about AI capabilities and impacts on lives and livelihoods. This lack of awareness may be attributed to efforts to mystify AI to circumvent the need to explain complex technologies and create a sense of awe about AI capabilities (Campolo and Crawford, 2020). As a result, societies are ill-prepared for the impending AI revolution (Samuel, 2022). This trend of mystifying AI to the non-expert audience must be replaced with proactive education and training (Gleason, 2018), where AI is centered around humans and human wellbeing in the future.

Therefore, we define human-centered AI (HAI) as consisting of the principles, practice and information management of AI development and deployment with the foremost goal of ensuring human wellbeing, progress, safety and satisfaction. Fortunately, a shift in focus from a technology-centric approach to one that prioritizes people (Bingley et al., 2023) suggests that HAI is evolving in the right direction. HAI will be critical to ensure sustainable human engagement with AI (Shneiderman, 2020).

### Why consider AI education separately?

Hype about AI capabilities is matched by fears about its influence, effects, and unintended consequences. We discuss a few examples as we make a case for AI Education below.

First, there is considerable uncertainty about the cumulative systemic risks posed by AI, especially since it is rapidly changing the ways in which financial institutions and markets operate and are regulated. An increased use of AI technologies has raised the specter of unstable markets due to an overreliance on algorithmic trading and potential failures of regulatory policy. In addition, this has given rise to fears of systemic instabilities and mass unemployment if AI replaces humans in future organizational workforces (Daníelsson et al., 2022).

Second, there is a widespread fear that AI can be leveraged to invade privacy and foster a culture of surveillance. Researchers fear an assault on individual privacy and rights as AI enabled surveillance grows at an alarming pace (Bartneck et al., 2021). In addition, unintended consequences may arise from the sale and use of private data by companies for non-intended purposes.

Third, there is a fear that algorithmic biases can accelerate lack of access and opportunities to disadvantaged populations and create unintended consequences. Algorithmic biases such as dataset bias, association bias, automation bias, interaction bias, and confirmation bias, amplify social biases (Chou et al., 2017; Lloyd, 2018). Software and technological implementations may contain implicit biases from developers or from the development ecosystem (Baker and Hawn, 2021). For instance, natural language processing (NLP) applications are known to amplify gender bias and Automated Speech Recognition (ASR) technologies have been found to display racial bias (Mengesha et al., 2021). Algorithmic bias can lead to multiple deliberate and unintended consequences such as discrimination, biased outcomes, and a lack of transparency and knowledge about how AI is involved in decision outcomes (Mikalef et al., 2022).

To assuage these fears and counter these challenges, we must develop new multidisciplinary and interdisciplinary frameworks (Touretzky et al., 2019; Chiu et al., 2021) to devise strategies to counter AI-induced risks and challenges and impart AI education globally.

However, AI education poses several unique challenges. Teaching AI topics across diverse cultural contexts can be daunting because the ability and willingness to learn often varies across cultures. Research has shown that cultural ecosystems, which impose their own systemic needs, play an important role in influencing their members' willingness to adopt new technology. Cultural differences between nations and people have an impact on how technology is adopted as some cultures are less open to new ideas than others (Tubadji et al., 2021). As a result, educators may be required to teach students who are resistant to learning AI because they either distrust or fear AI, or perceive AI technology to be counter to the norms of their cultures. Further, there is a growing perception that AI-related research is predominantly undertaken by the WEIRD (White, Educated, Industrialized, Rich and Democratic) countries, displaying cultural imbalances in AI research (Henrich et al., 2010; Schulz et al., 2018; Mohammed and Watson, 2019). This suggests that AI research often overlooks the distinct challenges of implementing AI in cultures beyond the predominant ones found in WEIRD countries. Therefore, it is imperative to foster greater inclusivity in AI research, while prioritizing the urgent need for AI education that is sensitive to cultural context.

## Development of the CATE-AI framework

### What is culturally responsive teaching?

Culture encompasses elements such as language, beliefs, values, norms, behaviors, and material objects, which are transmitted from one generation to the next. Additionally, it's important to note that every individual worldwide belongs to at least one culture (Skiba and Ritter, 2011; Bal, 2018). Individuals are impacted by the values upheld within specific cultures, resulting in differing levels of receptiveness toward AI e. Bal (2018) concisely expressed culture's substantial effect on human nature, arguing that culture pervades all aspects of human existence. This underscores the importance of culturally sensitive AI design to achieve optimal AI adoption and engagement.

Despite previous attempts at technology-based solutions for cultural and learning style mapping, there are currently few effective AI applications that can guide the selection of learning models tailored to specific learning contexts (Bajaj and Sharma, 2018). For instance, in the field of human computer interaction, experts agree that a User Interface (UI) design that meets the preferences, differences, and needs of a group of users can potentially increase the usability of a system (Alsswey and Al-Samarraie, 2021). However, the widespread adoption of exclusionary AI, such as Automated Speech Recognition (ASR) technologies that frequently exhibit racial bias, demonstrates the lack of progress in the development of intuitive and culturally-sensitive AI (Mengesha et al., 2021). Further, the underrepresentation of non-dominant communities in AI research exacerbates bias due to the imbalance between the volume of research conducted in WEIRD nations vs. non-dominant communities.

To develop more inclusive and intuitive AI technology, it is critical to impart quality AI education using personalized learning approaches (Chassignol et al., 2018). Culturally Responsive Teaching (CRT) is a personalized framework sensitive to the learner's cultural context.

The CRT framework utilizes the cultural knowledge, past experiences, reference points, and performance of students from diverse ethnic backgrounds to enhance the relevance and effectiveness of their learning experiences (Gay, 1993, 2002, 2013, 2021). CRT guides instruction by emphasizing and leveraging their inherent strengths. It supports their behaviors, knowledge, beliefs, and values, while acknowledging the significance of racial and cultural diversity in the learning process. CRT's intuitive appeal results from its flexibility to its manifestation in various forms, each with its distinct shapes and outcomes. Due to its intuitive appeal, CRT empowers educators to embrace the framework and integrate its principles into teaching diverse groups of learners.

### Educational AI

There are several advantages to leveraging Artificial Intelligence (AI) technologies in education. AI can be an effective tool for formulating personalized instructional systems. Such AI-supported systems can promote exploratory learning via dialogues, analyze student writing, simulate game-based environments with intelligent agents, and resolve issues with chatbots. AI can also facilitate student/tutor matching, putting students in control of their own learning (Holmes et al., 2020). This can be especially helpful for students who are at a disadvantage in learning due to teaching styles or lack of physical access to school. However, establishing digital infrastructure and providing access to digital learning platforms will be necessary for this to work.

There are two ways in which AI technologies can improve the quality of education. First, AI can support educators by improving efficiency in the performance of administrative tasks, such as reviewing student work, grading, and providing feedback on assignments through automation using web-based platforms or computer programs (Chen et al., 2020). This can enable educators to devote valuable time to focus on research and improve their teaching methodology and content. In the long-term, this can also help in improving the mental health of educators. Second, AI can support learners by customizing and personalizing curriculum and content in line with learners' needs, abilities, and capabilities (Mikropoulos and Natsis, 2011). By analyzing performance data, AI can identify gaps in knowledge and tailor instructional materials and resources to better suit individual learners. This can help students learn at their own pace and in a way that suits their learning style, leading to better academic performance and increased motivation to learn.

### Importance of cultural intelligence

In today's globalized world, effective cross-cultural communication is becoming increasingly important. As a result, the concept of cultural intelligence is gaining considerable traction. Moreover, while previous emphasis has been on developing Intelligence Quotient (IQ) and Emotional Quotient (EQ), cultural intelligence is a newer concept that builds upon emotional intelligence (Van Dyne et al., 2010). Emotional intelligence (EI) involves the ability to carry out accurate reasoning about emotions and the ability to use emotions and emotional knowledge to enhance thought (Mayer et al., 2008). Cultural intelligence, or cultural quotient, takes this a step further by emphasizing the importance of understanding and adapting to different cultural contexts. As a result, there is a substantial push to impart cultural knowledge among those in leadership positions in order to improve productivity. However, it is important that cultural intelligence is not only emphasized in the corporate sector, but also in education, which is the foundation of society (Earley and Mosakowski, 2004).

To be successful in today's diverse and interconnected world, educators must also be able to effectively navigate and communicate with individuals from different cultures. This requires cultural intelligence, which refers to not only a basic understanding of different cultural norms and practices, but also the ability to adapt one's behavior and communication style to different cultural contexts. Cultural intelligence encompasses more than just knowledge about different cultures; it also includes the ability to build relationships with people from diverse backgrounds and effectively collaborate with them. Van Dyne et al. (2010) have developed a four-factor model of cultural intelligence that can be effective in developing cultural intelligence in the field of teaching in an AI-powered world. This model includes Motivational Cultural Quotient, which refers to a leader's interest, drive, and energy to adapt cross-culturally; Cognitive Cultural Quotient, which involves a leader's cognitive understanding of culture; Metacognitive Cultural Quotient, which involves their ability to strategize across cultures; and Behavioral Cultural Quotient, which provides the ability to engage in effective, flexible leadership across cultures.

In an AI-powered world, it is becoming increasingly crucial for educators to leverage cultural intelligence. As the world becomes more interconnected, educators need to be equipped with the ability to navigate diverse cultural contexts, communicate effectively with individuals from different backgrounds, and collaborate with them. It is important for educators to develop the necessary skills to succeed in this globalized landscape, adapting their teaching style and creating inclusive learning environments that embrace diversity and promote cross-cultural understanding.

### Philosophy for AI and human interaction

A clear philosophical perspective is needed to frame the importance of AI and AI education. It is only with a well-integrated philosophical perspective that we can weave AI ethics, sociocultural priorities and sensitivity to humans into AI development and deployment. AI philosophy is critical for the sustainability of AI as a science. At the core of the philosophy of AI and human interaction, is the research driven belief that AI can augment human intelligence, if applied correctly, and enhance human performance to optimal levels using adaptive cognitive fit mechanisms (Samuel et al., 2022b).

Furthermore, as the development of virtual agents increasingly focuses on the social aspects of human interaction, it becomes crucial to address the notion of culture and its impact on human behavior (Mascarenhas et al., 2016). By doing so, greater equity in the field of AI can be achieved, leading to inclusive outcomes such as a more equitable representation of people from diverse contexts in AI, along with greater willingness to use AI technologies. It is important to distinguish between teaching AI as a subject and using AI as a tool to teach other subjects. AI education does not happen in a vacuum; rather, it takes place in diverse settings where learners from different cultural backgrounds are present. Cultural context plays a crucial role in shaping the ability and willingness of learners to engage with AI education.

Algorithms often embed designer or societal biases, resulting in discriminatory predictions or inferences (Baker and Hawn, 2021). Consequently, ensuring the representation of people from different cultures is crucial in developing AI technologies that can be used globally and across cultures. However, AI education itself is not inclusive for all cultures. Research has shown that cultural ecosystems, which impose their own systemic needs, play an important role in influencing their members' willingness to adopt new technology. Another important factor contributing to an individual's attitude toward AI technologies is gender (Horowitz and Kahn, 2021). “Because some cultures are less receptive to new ideas than others, cultural differences across countries and individuals influence the adoption of technology” (Tubadji et al., 2021).

### The CATE-AI framework

There are several ways in which cultural differences manifest when it comes to learning about AI. Gender, which typically cuts across cultures, disadvantages people who do not identify as men. There is frequently a lack of gender diversity in the field of AI (Samuel et al., 2018). This is partially a result of the manner in which AI is taught in educational institutions. In schools, AI curriculum is typically offered as part of computer science and STEM subjects, which are science, technology, engineering, and mathematics. However, AI instruction has often been conducted after formal lessons and outside of regular classroom settings. As a result, the subject suffers from a lack of diversity, with most participants being high-achieving boys (Xia et al., 2022). This lack of diversity creates challenges related to inclusion and equity. Instances such as these underline the need for designing AI curricula which are sensitive to the needs of learners coming from different cultural contexts.

We used the aforementioned theories and concepts with the goals of HAI, and developed a framework to facilitate AI learning delivered in a way that is contextualized to social, cultural, individual and future workplace factors and needs: Culturally Adaptive Thinking in Education for Artificial Intelligence (CATE-AI) framework (Figure 2). These concepts are fundamental to the conceptualization, understanding and application of the CATE-AI framework: the foundational ideas and theoretical drives of CRT and cultural intelligence, the critical role of philosophy in framing the interaction between artificial and human intelligences for culturally sensitive creative thinking on AI, the importance of factoring the multidimensional aspects of human behavior to facilitate sustainable ease of use of AI adapted to culture and context, the increasingly powerful role of AI models and AI applications in human society and the need to repurpose and adapt focal AI artifacts to culture, context and geography, the cultivation of HAI through AI education which embodies persuasive reflections of repurposed and adapted AI artifacts in curricula, and the co-evolution of all of these with the emerging dynamics of the human centered AI ecosystem. Information and perceptions about AI play a critical role in shaping public opinion about AI and this is undoubtedly an influential force, which we consider as being an uncontrollable part of the HAI ecosystem, without a condition of alignment with the goals of HAI. The CATE-AI framework therefore posits five principles:

1. AI education needs to be developed with theoretical and philosophical foundations which specifically address human behavior, technological capabilities and human centered AI.
2. AI education needs to adapt to sociocultural contexts and demonstrate resilience against AI biases and limitations.
3. Culturally adaptive thinking is expected to lead to richer learning experiences and enhanced interest in the study and application of AI.
4. AI education must remain adaptive to changes in mutually influencing forces of AI technologies and human behavior.
5. Human intelligence and AI interaction philosophical foundation are expected to guide ethical, human values based and human centered AI development, deployment and education.

The CATE-AI framework can be applied to influence multiple levels of education and learning of AI. It can be used in developing curricula, learning management systems design, course and program effectiveness evaluation and also in measuring student satisfaction outcomes. The CATE-AI framework could serve as a valuable framework for informing faculty and those involved in course delivery–the adaptive “thinking” in CATE-AI refers to a mindset that applies to those who teach and to those who learn.

### Review of cases and discussion

In addition to theorization, we explored two sets of data qualitatively: the first is a set of outputs generated by GPT 3.5 Turbo based OpenAI application ChatGPT and the second consist of a collection of new headlines on AI from 2022 to 2023 (Figure 3). Our objectives were to explore the presence of bias and limitations in ChatGPT output, and to explore emerging and dominant societal themes on AI as reflected in news headlines and their influence on HAI initiatives. While we had nothing new and interesting to report in this study, our review of ChatGPT output confirmed numerous biases and limitations (Azaria, 2022; Borji, 2023). Furthermore, a review of news articles from around the world revealed that responses to AI, NLP, LLMs and applications such as ChatGPT varied significantly by region and culture. ChatGPT apparent biases related to religions, political ideologies, gender and race were observed (Figure 3).

**Figure 4:**
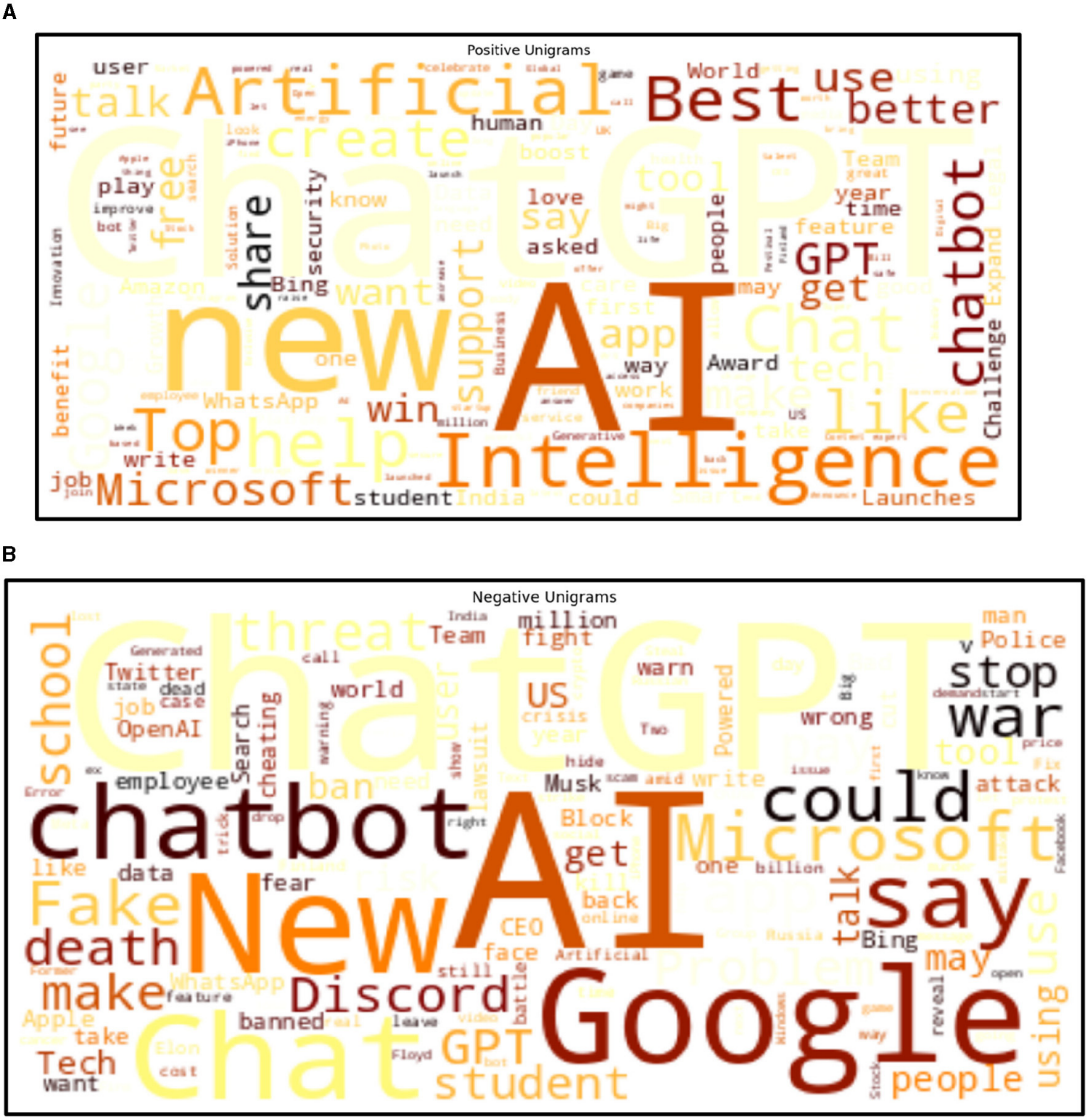
**(A)** Positive sentiment word cloud. **(B)** Negative sentiment word cloud.

**Figure 5:**
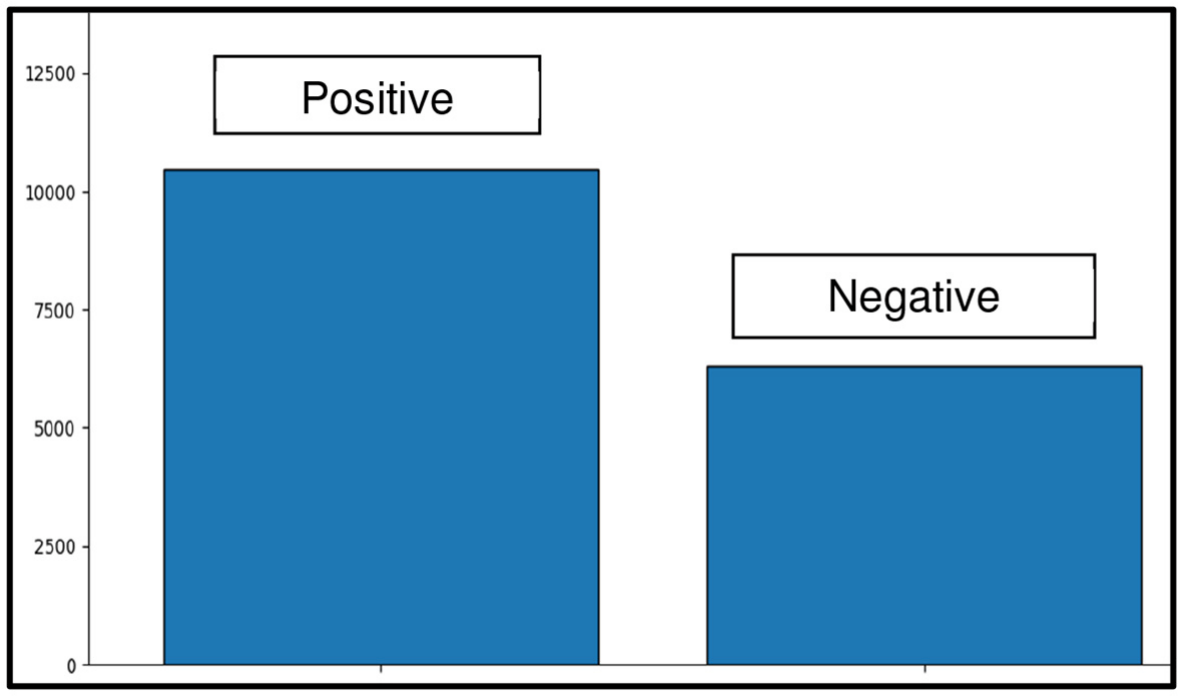
Positive to negative sentiment comparison.

**Figure 6:**
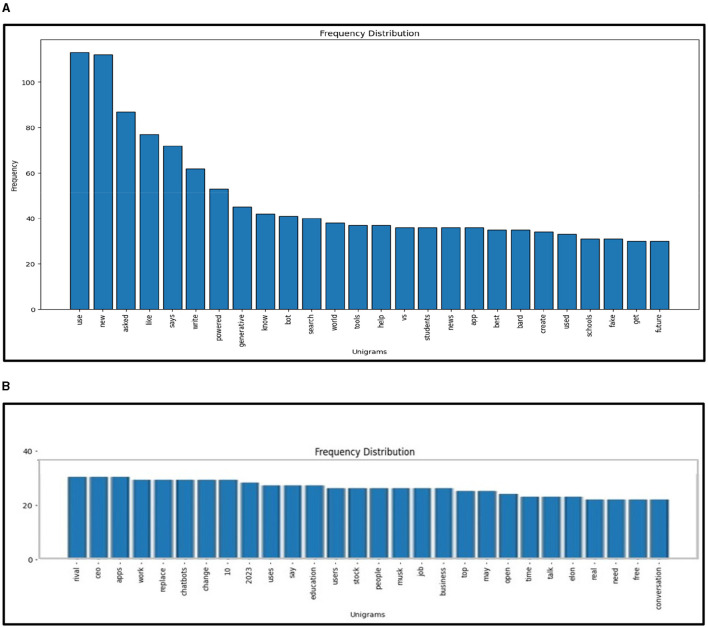
**(A)** Unigrams (part-1) of AI-ChatGPT news articles. **(B)** Unigrams (part-2) of AI-ChatGPT news articles.

Applying NLP methods, we also explored and summarized news headlines or articles relevant to AI, NLP, LLMs and ChatGPT by sentiment, and categorized them into positive, neutral and negative categories (Figures 4A, B, 5). We used NLTK for sentiment analysis (Bird et al., 2009). In addition to examining specific cases to motivate and inform CATE-AI, we performed exploratory analysis by visualizing word clouds from the text of the news headlines we collected from around the world. We also explored unigrams as visualized in a split manner in Figures 6A, B. Common and expected words such as “ai” and “gpt” were excluded from the unigrams to highlight the array of other high frequency words.

A review of the unigrams showed some interesting emphasis on topics in the conversations on AI, and some of these can be interpreted as being associated with most recent developments at the time of data collection, while other can be treated as being more persistent themes. We identified a fair amount of interest in technological developments with key words such as “generative,” “powered,” “bot” and “tools,” in concerns and risks of the future of AI with key words such as “fake,” “bot,” “future” and “rival,” in unusual focus on entities such as “Musk” and “Elon,” and in an emphasis on workplace themes with key words such as “use,” “new,” “job,” “write,” “create,” “ceo” and “business.”

**Figure 7:**
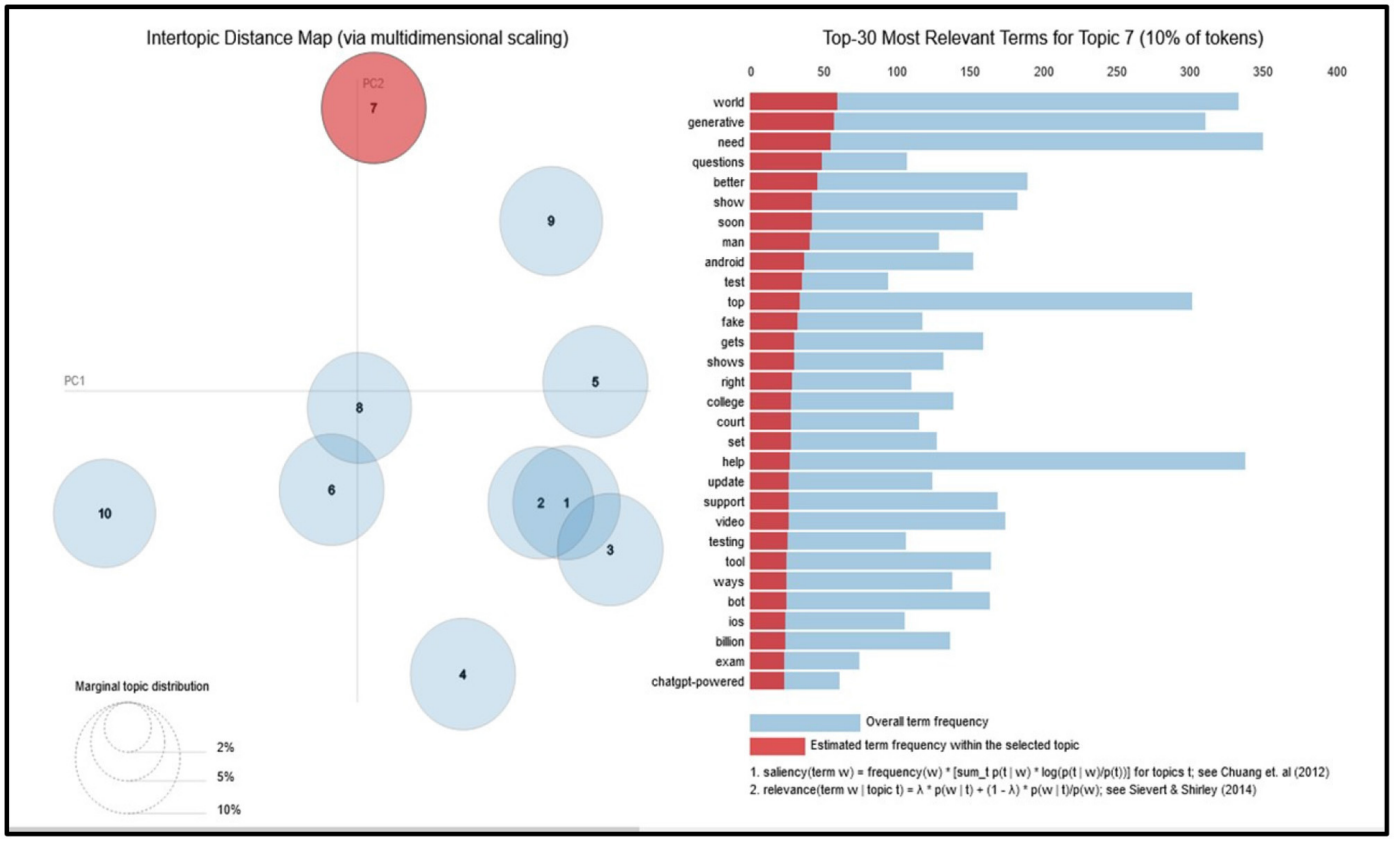
GENSIM LDA intertopic distance map.

Our NLP based analysis of the text revealed useful insights. Positive headlines outnumbered the negative ones at around a 5:3 ratio (Figure 5). The presence of such a notable number of negative headlines highlights the significant number of unaddressed concerns, issues and examples of AI failures and misses. The negative sentiment word cloud (Figure 4B) highlights high frequency words which were clustered to identify dominant themes such as concerns surrounding the misuse of AI for deception (fear, fake, wrong, cheating, banned, scam, trick), issues in education (school, student, cheating, test), jobs (job, employee, lawsuit, crisis) and security (block, warning, dead, war, battle, fight, police, threat). Additional bigram, trigram and quadgram analyses revealed similar patterns of word frequencies, including regional headlines with the implication of banning ChatGPT, using generative AIs unethically, emergence of fake AI apps and concerns around the impacts of AIs on human life, jobs and society (Samuel, 2023c). Some of these were identified as regional or ideological themes illustrating the need for CATE-AI, such as “collapse creative process,” “woke ChatGPT accused,” “AI arms race,” “ChatGPT passed Wharton MBA,” “students using AI” and “destroyed Google business.” We validated our findings on thematic topics by using the GENSIM Latent Dirichlet allocation (LDA) model with iterations of intertopic mapping and review of topics by key words associated with the topics (Rehurek and Sojka, 2010, 2011). We ran multiple iterations by removing disruptive and overbearing key words to evocate and affirm underlying themes and topics. The visualization of one such iteration is displayed in Figure 7, and this iteration lent support to the themes on concerns regarding the use of generative AI and AI in general for education (“college,” “exam,” “test,” “testing” and “questions”) and misuse (“fake,” “court” and “bot”). This process affirmed the dominant themes initially identified through exploratory textual analytics using word clouds and n-grams as described in the sections above.

We identified several interesting themes revolving around fear of AI, need to regulate AI, sociocultural challenges of AI applications, AI bias, limitations and inaccuracies in AI output, how-to topics, and unfairness-such as in comparison of religions. The variations in these themes observed across different regions and cultures underscore the importance of utilizing CATE-AI to create culturally sensitive AI education frameworks and curricula. CATE-AI can help address the challenges of sociocultural differences by guiding the adaptation of categorization frameworks of human-thought clusters (Murphy and Medin, 1985; Barsalou, 1989). Ideally, all relevant education initiatives should help learners understand four critical aspects of AI:

1. What is AI–a philosophical foundation for engaging AI.
2. How AI works–the science of AI technologies.
3. AI contextualization-how AI can be adapted to sociocultural contexts.
4. Optimal AI–using AI to support adaptive cognitive fit (Samuel et al., 2022b).

This will help prepare humans face a future filled with ubiquitous AIs through systematics AI literacy. CATE-AI can also help facilitate AI adaptation to sociocultural contexts across the world, with the potential to improve ease of use through informed use and user satisfaction outcomes.

## Limitations and future research

Our study provides robust conceptualization and theorization to advance the CATE-AI framework. However, additional work is needed to elaborate its tenets and develop applied solutions. However, we still need to conduct experiments and analyze implementation cases for CATE-AI to strengthen and further validate the framework. We caution that due to the exploratory nature of our analysis, we applied stop-words, including custom stop-words of common but non-insightful words (e.g., company names), to the textual data corpus to generate word clouds, sentiment analysis, and intertopic maps using LDA modeling (Figures 4A, B, 5, 7). Our analysis revealed the need for a dedicated study of this textual data corpus with additional NLP methods including clustering for topic and theme identification, information retrieval, named entity recognition (NER) and sentiment analysis to gauge sentiment toward AI applications by region using domain-knowledge bases (Kumar et al., 2022, 2023). These are avenues for future research and further intrinsic development of CATE-AI. Numerous extensions and applications of CATE-AI are possible, such as the extending its principles to AI generated multilingual solutions for making adaptive sense of human language and emotions across languages, regions and cultures (Anderson et al., 2023). Further, in many cultures, handwritten text is an essential part of educational and societal processes, and it will be interesting to see how AI tools for OCR can be adapted for better HAI design (Jain et al., 2023).

## Conclusion

Our research addresses a critical concern regarding an issue of global importance with significant implications for the future. AI cannot be paused or stopped. The extraordinary speed at which AI models and applications are being developed presents complex challenges and new opportunities (Samuel, 2023a,b). AI as a cluster of powerful transformative technologies has the potential to shape the future of human society. Therefore, AI education is of utmost importance and urgency for sustainable and equitable advancement. AI education is no longer optional and any proactive approach toward maximizing the benefits of AI and minimizing the risks and harms of AI must include a proactive approach to adaptive AI education (Samuel, 2021a). Insensitivity toward sociocultural needs and HAI education can lead to a rise in AI illiteracy, a growth in negative public perception, and heightened resistance toward AI. CATE-AI provides a futuristic framework to catalyze sensitive, fair and relevant AI education to the masses. Ideally CATE-AI must be co-implemented with proactive policies for mass-public education implementation initiatives based on continuing-education models. As we look forward to reaping the benefits of AI, we anticipate that CATE-AI and other AI educations models will play a crucial role in shaping the future.

## Data availability statement

Data used in this study can be found online, as schematic sample, via the following link: https://github.com/ay7n/CATE.

## Author contributions

All authors listed have made fair intellectual contributions to the manuscript and approved it for publication.
